# Bringing rigor in contextual objectivity: lessons from applying feminist lens in scoping the evidence on girlhood studies in Indonesia

**DOI:** 10.3389/frma.2024.1339651

**Published:** 2024-05-30

**Authors:** Santi Kusumaningrum, Shaila Tieken, Andrea Andjaringtyas Adhi, Siti Ainun Nisa, Widi Laras Sari, Annisa R. Beta

**Affiliations:** ^1^Center on Child Protection and Wellbeing, PUSKAPA, Universitas Indonesia, Depok City, Indonesia; ^2^School of Culture and Communication, University of Melbourne, Melbourne, VIC, Australia

**Keywords:** girlhood, feminist methodology, scoping review, advocacy, Indonesia, girls, young women, critical study

## Abstract

This perspective paper contemplates the nuances of engaging with literature ethically in conducting a scoping review based on the researchers' project on girlhood studies in Indonesia. We assert that the ethical perspective extends beyond conventional primary data collection from human participants, further emphasizing the essence of a feminist methodology in this scholarly investigation. We discuss the interplay between the role of rigor and the dynamics of power relations in research, shedding light on reconciling between the pursuit of facts and acknowledgment of biases in knowledge production. This reflection offers insights into the methodological process and the researcher's role, contributing to the broader discourse on how research can effectively address issues of gender equity and social inclusion. Through this paper, we underscore the necessity of an intentional approach in unifying the domains of science and advocacy because only then can we truly catalyze transformative change. In doing so, we seek to foster a more comprehensive, objective, and empathetic understanding of the researched: in this case, the experiences of girls and young women –and, by extension, marginalized individuals in Indonesia and beyond.

## 1 Introduction

This paper delves into the experiences the researchers encountered while conducting a scoping review on girlhood studies in Indonesia since June 2022. The review was designed to understand the extent to which nuances and complexity of girlhood have been afforded to the lives of girls and young women in Indonesia through how they have been researched. Based on our reflection on our scoping review for the Indonesian girlhood study, we advance a term called “contextual objectivity” to capture the importance of paying attention to the landscape in which a specific study is located as well as the nuance within the study. We argue that contextual objectivity is important in scoping studies in discerning and exploring the semantics and the latent in the literature we gathered. Beyond the breadth and depth of a study area, we believe that a scoping review can have a greater role in unpacking how topics emerged, discussed, and linked with each other. Applying contextual objectivity in the review allows us to investigate how publications present Indonesian girls and problematize phenomena around their lives.

The application of contextual objectivity in the scoping review process was motivated by a feminist lens that emphasizes the importance of reflexivity, inclusivity, and the recognition of power dynamics in research. Feminist methodology involves engaging with the situatedness and contextually embedded nature of data, centering the relationship between the researcher and the researched, and valuing marginalized voices (Millen, 1997; Norgbey, 2018; Mootz et al., 2019). Feminist qualitative research often employs methods such as interviews, focus groups, participant observation, and content analysis to explore the experiences and perspectives of individuals and communities (Chilisa and Tsheko, 2014; Morgan et al., 2018; Karimi, 2019). Emotionally engaged researchers, in the case of this scoping review, describe not only engagements with research participants but also literature (Campbell, 2001). These forms of reflection, where experiences converse with interpretations, are acknowledged to enrich and reorient the review processes (Blakely, 2007).

In the next section of this paper, we will introduce how the feminist approach was implemented in our scoping review. The third section of the paper will present the researchers' reflections through the scoping review process. Lastly, we will discuss our reflections thoroughly in section four.

## 2 Applying the feminist approach in a scoping review

This article presents our reflections gained from applying the feminist lens on the scoping review method, a technique for mapping and synthesizing the evidence from scholarly works within a specific field that was first introduced in the context of delivery and organizational health services (Mays et al., 2001). A scoping review is a type of systematic approach that provides an overview of the existing literature, identifies key themes, trends, and gaps, and allows more flexibility for iterative refinement of the research question and inclusion criteria (Arksey and O'Malley, 2005). Since the scoping review is based on a traditional approach to evidence-based practice, it may be limited in its search for definitive answers and in extracting deconstructed information out of context. Although scoping reviews are becoming more common, they have been predominantly used in the health sector rather than in social sciences (Pham et al., 2014).

The proponents of feminist methodologies have criticized the conventional understanding of knowledge, which often privileges objective, detached viewpoints and often overlooks the importance of subjective experience and social context (Code, 2013). In addition, the traditional approach to synthesizing knowledge tends to focus on looking for patterns and similarities, overlooking what she describes as “not knowing” or the unknown context surrounding the generalized pattern (Lather, 2009). In conducting the scoping review, we observe the limitations of the traditional method raised by proponents of feminist methodology. We also note potential biases of the researchers and the knowledge production environment in which the literature was produced. Moreover, we also question the granularities that are sacrificed to create generalization and categorization and attempt to figure out spaces for the contributions of the smaller threads of analysis.

Therefore, in our exercise, we aim to reconcile the scoping review's methodological limit by applying a feminist approach as we re-examine our roles and positionality as researchers, our intentions, and the material effects of our research (Saraswati and Beta, 2021). Beyond charting the scholarly landscape of girlhood studies in Indonesia, the review tries to untangle the conflated views and assumptions on Indonesian girls internally within its own field and externally, among other girlhood studies in the global south.

## 3 Conducting a scoping review on critical girlhood studies in Indonesia

Critical girlhood studies provide a transformative lens, challenging the predominant focus on white, middle-class girls by inviting a nuanced and engaged exploration of the perspectives of girls from diverse backgrounds, critically investigating and celebrating their joy of being outside the usual epistemic centers (Bae-Dimitriadis, 2017) and their ability to flip the script and shapeshift in order to negotiate the opportunities and limitations in their everyday lives (Rentschler and Mitchell, 2014; Cox, 2015; Evans-Winters, 2017). Brown (2011) argued that critical girlhood studies aim to “problematize all the assumptions couched under the term, expose them to scrutiny, and address their politics, all the while maintaining the complexities of identity that the contested label appropriately reflects” (109). Assessing the scope of girlhood studies in Indonesia is our effort to contribute to the critical discussion of girlhood studies. Emerging in the past two decades as a subfield in youth studies, it has successfully opened the pathway for research projects and academic discussions that are critical of how girls and girlhood have been constructed by including perspectives from feminist media studies, gender studies, queer studies, critical race studies, critical development studies, cultural studies, and youth sociology (McRobbie and Garber, 1976; Harris, 2004a,b, 2008; Gonick et al., 2009; Kearney, 2009, 2011, 2018; McRobbie, 2009; Taft, 2010, 2014; Renold and Ringrose, 2013; Gill and Orgad, 2015; Mendes et al., 2019).

Nevertheless, the studies generated in critical girlhood studies remain Western and Global North-centric. It leaves studies about girls in the Global South generally and in Southeast Asia riddled explicitly with overdetermination and simplification of their cultural, social, and political contexts (Bae-Dimitriadis, 2017; Khoja-Moolji, 2018; Loh, 2020; Saraswati and Beta, 2021) -generating most analysis on girls in the region with a marked “third world difference” (Mohanty, 2003). We try to fill the field's gap in studying global south “girls as doers,” which shifts the gaze from the “crisis of girlhood” to girls' engagement and participation (Mitchell, 2016). We offer our viewpoints about positioning ourselves as engaged scholars while scrutinizing the contexts in which studies on Indonesian girls were designed, published, and used.

We believe that the feminist lens should be integral to our effort to chart girlhood studies in Indonesia critically. Thus, reflexive thinking was employed to ensure rigor in our scoping review and applying the feminist approach (Rettke et al., 2018). To achieve the objective of the scoping review with a feminist lens, we systematically allocated time and space for reflection to discuss project progress and the rigor at each review stage. Consisting of women of various ages and backgrounds, the team finds the importance of creating space to discuss their thoughts and reflections during each review stage and also giving nuances in each own experience as a girl. The team recorded all decisions and reflections in a working memo, typically written by a team member acting as a note-taker. The purpose of this memo is to encourage team members to record their interpretations and reflections throughout the analysis process, similar to an all-access diary. The reflective memo also provides a space for researchers to practice discipline in being reflective while challenging the researcher's biases in understanding how girls were discussed. Each stage of the scoping review is rigorously completed while applying a feminist lens and practicing reflexive thinking in conducting the scoping review.

Reflections allow researchers to delve into the data without losing the context in which the study was conducted. One such reflection occurs when we start categorizing the descriptive information about the literature, including its study population. At the onset of our study, we gathered the literature from Google Scholar, retrieving 18,793 academic publications from Indonesian and international journals. Early in our screening, we noticed that Indonesian publications are not always indexed internationally, pushing us to step back and scrutinize the quality of Indonesian papers. Through our reflection sessions, we recognized that selecting Indonesian journal articles also revealed the barriers Indonesian researchers might experience in publishing their studies, and thus increased our awareness of the politics of knowledge production in girlhood studies. In our charting stage, we tried to capture both studies around “girls,” defined as individuals under 18 years of age, and studies including “girls” and young “women” broadly defined. We took the decision to glean into girlhood as defined by Indonesian scholars without the constraints of normative definitions. As a result, our study captured literature involving girls within various age brackets, which was challenging to categorize. Although we aimed to identify major commonalities through categorization, we realized that the age range in this study is contextual and needs to be interpreted beyond the numbers it represents. We decided to omit the coding for categorization for age and, instead, learn when, where, and which girls are part of women from different age groups.

Furthermore, in engaging with the literature, we employed a diagramming method to conduct thematic analysis and discern the patterns and relations in the texts. At this stage of the review, we realized that the way the researchers charted the data and interpreted the literature was not always aligned. We started reflecting on our choice of words in producing themes and what meaning they derive from our background and experiences. For example, when we try to find the thematic threads in “Who” the girls are in the literature, our perception of how the authors represent girls and our own interpretation collides. The researchers are found to interpret adverse experiences, such as early marriages, as victimhood or victimizations without a clear account in the respective literature on the girls' position. Acknowledging the bias in analyzing the data has been an important step for researchers in every discussion. In doing our research, we also consciously take a step back and observe our ways of identifying girls' experiences. One of the researchers reflected: “*I noticed that one literature could discuss more than one dimension and representation of girls. For example, in cases of teenage pregnancy/child marriage, girls could be discussed within their roles as “adolescents,” but at the same time, they could also be depicted within their roles as “mothers,” “wives,” or “victims.”* As literature can convey girls' multitude of roles, researchers' decisions in encapsulating the myriad of terms for the convenience of analysis should be carefully assessed and constantly reflected.

## 4 Discussion

*This is how empathy violates the other and is part of the demand for totality. A recalcitrant rhetoric is about inaccessible alterity, a lesson in modesty and respect, somewhere outside of our desire to possess, know, grasp (Lather*, *2000**)*.

This paper captures our insights in applying the principles, employing a “contextual objectivity” and feminist lens in striking a balance between the researchers and the subjects we read in the literature on other people's experiences. The research team comprises women from diverse backgrounds and training, including criminology, cultural studies, economics, public health, and psychology. While all of us are working in the field of gender and child protection, we found our experience affects the lens through which we are observing the literature and the girls they discussed. As one of the researchers with a criminology background reflected: “*In the first stage of our inventorying, I realized that we must be critical in treating the subjects as ‘victims.' We tend to categorize individuals as victims when they are on the receiving end of harmful treatments, but they were not always presented that way in the literature. Thus, it is necessary for us to define what ‘presented as victims' means.”*

### 4.1 Reconciling scholarship and activism

Feminist methodology also plays a crucial role in reconciling science and advocacy. It recognizes the interconnectedness of research and activism and seeks to bridge the gap between theory and practice (Fox and Murry, 2000). By engaging in research that is politically and ethically accountable to disadvantaged groups, feminist methodology promotes social change and challenges existing power structures (Gurung, 2021). It provides a framework for researchers to navigate the complexities of conducting research that is both rigorous and socially transformative (Harbin, 2014).

Through our process of investigating how the literature defined problems, challenges, and opportunities influencing Indonesian girls, we recognized the tension between scholarship and activism. For us, applying the feminist approach is more than applying feminist rigor in our review. As women and policy researchers, we understand our own biases when discussing the positions of Indonesian girls in the literature. We recognize the significant role we play as advocates for girls throughout every stage of the scoping review. For example, during the screening stage, we use our judgment and advocacy position to include articles on important topics or from Indonesian journals, even if their quality may differ from top international journals.

### 4.2 Promoting ethical research practices, including in scoping review

Feminist methodology considers ethics by placing a strong emphasis on ethical conduct in research. It recognizes the power dynamics between researchers and participants and aims to address these power imbalances. Feminist researchers strive to create a research environment that is respectful, inclusive, and empowering for all involved (Ackerly and True, 2008; Ruan, 2020). Feminist methodology recognizes that ethics are applicable in both primary data collection involving humans and in indirect or secondary data analysis, including literature reviews. It acknowledges that engaging with existing literature requires ethical considerations, such as ensuring proper citation and respecting the intellectual property of authors (Grosser, 2009). Feminist researchers approach literature reviews with a critical lens, questioning the power dynamics and biases present in the literature and seeking to amplify marginalized voices (Rizvi, 2017).

Irwin (2013) explores the ethics and epistemology of qualitative secondary analysis and highlights the importance of engaging with the situatedness and contextually embedded nature of qualitative data (Irwin, 2013). It emphasizes the need for ethical conduct in secondary analysis and the importance of considering the original context and intentions of the data. In content analysis, feminist methodology ethics is applied by critically examining the power dynamics embedded in the data and the research process. It involves questioning the underlying assumptions and biases that may be present in the content being analyzed. Mauthner et al. (1998) also call attention to using secondary qualitative data in research, which will separate the interpretation from its contextual information. Considering the increasing reliance on the internet in research, we also must consider and interpret literature in our review within the context, biases, and ethical implications of online research (Hokke et al., 2018). In our review, we were committed to upholding ethical standards by actively addressing our interests in advancing gender equality and promoting social justice for girls and young women. Instead of sidelining these concerns, we embraced reflexivity as a guiding principle, engaging in continuous reflection and constructive dialogues among ourselves. Throughout this process, we remained vigilant in critically reassessing the power dynamics inherent in the politics of knowledge production, thus ensuring a nuanced and conscientious approach as we worked on the scoping review.

Applying contextual objectivity in our review involves a conscious attempt to record our pathways of thinking and reflections through a memo, where we record our reflections and interpretations by asking ourselves “what do we think and feel about the findings in our review?”, as well as responding or challenging other team members' opinions. We suggest that objectivity entails transparency of our positions and limits in knowing. Through this memo, we were able to record our pathway of thinking, such as the shift in our interpretation on the discussion of girls' adverse experiences, where at the screening stage, we reflect: “*most of the literature discusses them as victims because they experience discrimination and violence”*, and we could see a shift in our understanding where at the analysis stage, we acknowledged our bias from our experience as policy researchers, as one of the researcher's reflected: “*During the extracting of variables from our matrix reading, I realized that the definition of victim, vulnerable group, and at-risk are often used in answering our matrix variables. During our debrief after 30 min of extracting, it is important to note that the researchers need to look into what the literature means when talking about girls. I also acknowledge my bias based on my background and my recent works that I see through the lens of vulnerable people. At first, I see them all as vulnerable groups, as they have more risks to be disadvantaged. But then, I am learning to also learn some perspectives.”* In considering the ethical implications of our interpretations, we acknowledge our intentional bias to ground our analysis in a commitment to social justice and equality (Faulkner, 2018; Manda and Settler, 2018).

### 4.3 Key implications for future studies

While the literature specifically addressing ethical considerations in conducting literature reviews or secondary data analysis is limited, there is a growing recognition of the need for ethical conduct in research that does not involve primary data collection. Researchers are encouraged to consider issues such as transparency, privacy, confidentiality, and the original context of the data when conducting literature reviews or secondary data analysis.

Feminist methodology places a strong emphasis on ethics in research. It considers power dynamics, inclusivity, and the wellbeing of participants. It applies ethical considerations in content analysis by critically examining the data and ensuring a commitment to social justice. Feminist methodology recognizes the importance of ethics in primary data collection and engaging with existing literature, including literature reviews. By prioritizing ethical conduct, it aims to create a more equitable and just research practice.

The preliminary findings of our scoping literature review suggest that there is a tendency in contemporary studies of girls to frame girls and young women in locations like Indonesia as already victims and/or subjects of development. This is where feminist approaches play a key role in reminding and challenging the researchers in their critical roles as knowledge producers. Feminist “contextual objectivity” encourages us to prioritize rigor *and* kindness. It compels us to consider the politics of knowledge production at play. It demands that we do not take the positions of the researched in the literature at face value but rather pursue an inquiry on why girls and young women in the Global South are and continue to be in particular positions. In the future, we suggest that researchers interested in investigating the livelihoods of girls should reflect upon and question their own position and framing of girls when designing the study. Researchers are invited to reflect, challenge, and be mindful of the method used to answer the research questions when studying girls' livelihoods while acknowledging their strengths and limitations.

## Data availability statement

The original contributions presented in the study are included in the article/supplementary material, further inquiries can be directed to the corresponding author.

## Author contributions

SK: Conceptualization, Writing—original draft. ST: Writing—original draft. AA: Writing—review & editing. SN: Writing—review & editing. WS: Writing—review & editing. AB: Conceptualization, Supervision, Writing—review & editing.

## References

[B1] Ackerly B. True J. (2008). Reflexivity in practice: power and ethics in feminist research on international relations. Int. Stud. Rev. 4, 693–707. 10.1111/j.1468-2486.2008.00826.x

[B2] Arksey H. O'Malley L. (2005). Scoping studies: towards a methodological framework, Int. J. Soc. Res. Methodol. 8, 19–32. 10.1080/1364557032000119616

[B3] Bae-Dimitriadis M. (2017). Introduction to the Special Issue on Girls From Outer Space: Emerging Girl Subjectivities and Reterritorializing Girlhood. Los Angeles, CA: SAGE Publications.

[B4] Blakely K. (2007). Reflections on the role of emotion in feminist research. Int. J. Qual. Methods 6, 59–68. 10.1177/16094069070060020634281104

[B5] Brown M. (2011). The sad, the mad and the bad: co-existing discourses of girlhood. Child Youth Care Forum 40, 107–120. 10.1007/s10566-010-9115-5

[B6] Campbell R. (2001). Emotionally Involved: The Impact of Researching Rape. New York: Routledge.

[B7] Chilisa B. Tsheko G. (2014). Mixed methods in indigenous research. J. Mix. Methods Res. 3, 222–233. 10.1177/1558689814527878

[B8] Code L. (2013). “Taking subjectivity into account.” in Feminist Epistemologies, L. Alcoff and E. Potter (New York: Routledge).

[B9] Cox A. M. (2015). Shapeshifters: Black girls and the Choreography of Citizenship. Durham: Duke University Press.

[B10] Evans-Winters V. E. (2017). Flipping the script: the dangerous bodies of girls of color. Cult. Stud. ↔ Criti. Methodol. 17, 415–423. 10.1177/1532708616684867

[B11] Faulkner S. (2018). Crank up the feminism: poetic inquiry as feminist methodology. Humanities 3, 85. 10.3390/h7030085

[B12] Fox G. Murry V. (2000). Gender and families: feminist perspectives and family research. J. Marriage Family 4, 1160–1172. 10.1111/j.1741-3737.2000.01160.x

[B13] Gill R. Orgad S. (2015). The confidence cult(ure). Aust. Feminist Stud. 30, 324–344. 10.1080/08164649.2016.1148001

[B14] Gonick M. Renold E. Ringrose J. Weems L. (2009). Rethinking agency and resistance: What comes after girl power? Girlh. Stud. 2, 1–9. 10.3167/ghs.2009.020202

[B15] Grosser K. (2009). Corporate social responsibility and gender equality: women as stakeholders and the european union sustainability strategy. Busin. Ethics Eur. Rev. 3, 290–307. 10.1111/j.1467-8608.2009.01564.x

[B16] Gurung L. (2021). Feminist standpoint theory: conceptualization and utility. Dhaulagiri J. Sociol. Anthropol. 14, 106–115. 10.3126/dsaj.v14i0.2735719999830

[B17] Harbin A. (2014). Mentorship in method: philosophy and experienced agency. Hypatia 2, 476–492. 10.1111/hypa.1208223607825

[B18] Harris A. (2004a). All About the Girl: Culture, Power, and Identity. London: Routledge.

[B19] Harris A. (2004b). Future Girl: Young Women in the Twenty-First Century. London: Routledge.

[B20] Harris A. (2008). Next Wave Cultures: Feminism, Subcultures, Activism. London: Routledge.

[B21] Hokke S. Hackworth N. Quin N. Bennetts S. Win H. Nicholson J.. (2018). Ethical issues in using the internet to engage participants in family and child research: a scoping review. PLoS ONE 9:e0204572. 10.1371/journal.pone.0204572 30261041 PMC6160098

[B22] Irwin S. (2013). Qualitative secondary data analysis: ethics, epistemology and context. Prog. Dev. Stud. 4, 295–306. 10.1177/1464993413490479

[B23] Karimi A. (2019). The role of intimate relationship status, sexuality, and ethnicity in doing fieldwork among sexual–racial minority refugees: an intersectional methodology^*^. Sociol. Inq. 4, 971–992. 10.1111/soin.12316

[B24] Kearney M. C. (2009). Coalescing: the development of girls' studies. NWSA J. 21, 1–28. 10.1353/ff.2009.a26365434409987

[B25] Kearney M. C. (2011). Mediated Girlhoods. Lausanne: Peter Lang. Available online at: https://www.peterlang.com/view/title/21140 (accessed November 4, 2023).

[B26] Kearney M. C. (2018). Girls' media studies. Femin. Media Histor. 4, 90–94. 10.1525/fmh.2018.4.2.90

[B27] Khoja-Moolji S. (2018). Forging the Ideal Educated Girl: The Production of Desirable Subjects in Muslim South Asia. California: University of California Press.

[B28] Lather P. (2000). “Against empathy voice and authenticity,” in Kvinder, Køn & Forskning.

[B29] Lather P. (2009). Getting Lost: feminist efforts toward a double(d) science. Front. J. Women Stud. 30, 222–230. 10.1353/fro.0.003234409987

[B30] Loh B. (2020). Tween girls' dressing: shifts, implications, and future directions in Singapore. Sociol. Compass 14:e12767. 10.1111/soc4.12767

[B31] Manda D. Settler F. (2018). Contestations of self and other in researching religion, gender and health among migrant women. Alternation 22, 119–141. 10.29086/2519-5476/2018/sp22a7

[B32] Mauthner N. S. Parry O. Backett-Milburn K. (1998). The data are out there, or are they? Implications for archiving and revisiting qualitative data. Sociology 32, 733–745. 10.1177/0038038598032004006

[B33] Mays N. Roberts E. Popay J. (2001). “Synthesising research evidence,” in Studying the Organisation and Delivery of Health Services: Research Methods, eds. N. Fulop, P. Allen, A. Clarke and N. Black.

[B34] McRobbie A. (2009). The Aftermath of Feminism: Gender, Culture and Social Change. Los Angeles, CA: SAGE.

[B35] McRobbie A. Garber J. (1976). “Girls and subcultures,” in Resistance Through Rituals: Youth Subcultures in Post-War Britain, eds. S. Hall and T. Jefferson (New York: Harper Collins Academic), 209–222.

[B36] Mendes K. Ringrose J. Keller J. (2019). Digital Feminist Activism: Girls and Women Fight Back Against Rape Culture. Oxford: Oxford University Press.

[B37] Millen D. (1997). Some methodological and epistemological issues raised by doing feminist research on non-feminist women. Sociol. Res. Online 3, 114–128. 10.5153/sro.1351

[B38] Mitchell C. (2016). Charting girlhood studies. Girlh. Polit. Place 2016, 87–103. 10.2307/j.ctt14jxn16.10

[B39] Mohanty C. T. (2003). Feminism Without Borders: Decolonizing Theory, Practicing Solidarity. Durham, NC: Duke University Press.

[B40] Mootz J. Muhanguzi F. Greenfield B. Gill M. Gonzalez M. Panko P.. (2019). Armed conflict, intimate partner violence, and mental distress of women in northeastern uganda: a mixed methods study. Psychol. Women Q. 4, 457–471. 10.1177/0361684319864366 35662739 PMC9165613

[B41] Morgan R. Ayiasi R. Barman D. Buzuzi S. Ssemugabo C. Ezumah N.. (2018). Gendered health systems: evidence from low- and middle-income countries. Health Res Policy Sys 1:5. 10.1186/s12961-018-0338-5 29980230 PMC6035473

[B42] Norgbey E. (2018). The role of feminist standpoint and intersectionality epistemologies in providing insights into the causes of gender disparity in higher education. EJRE 1, 19. 10.18192/ejre.v6i1.2063

[B43] Pham M. T. Rajić A. Greig J. D. Sargeant J. M. Papadopoulos A. McEwen S. A. (2014). A scoping review of scoping reviews: advancing the approach and enhancing the consistency. Res. Synth. Methods 5, 371–385. 10.1002/jrsm.1123 26052958 PMC4491356

[B44] Renold E. Ringrose J. (2013). Feminisms re-figuring ‘sexualisation', sexuality and ‘the girl.' Femin Theory. 14, 247–254. 10.1177/1464700113499531

[B45] Rentschler C. A. Mitchell C. (2014). The re-description of girls in crisis. Girlh. Stud. 7, 2–7. 10.3167/ghs.2014.070102

[B46] Rettke H. Pretto M. Spichiger E. Frei I. A. Spirig R. (2018). Using reflexive thinking to establish rigor in qualitative research. Nurs. Res. 67, 490–497. 10.1097/NNR.0000000000000307 30067583

[B47] Rizvi S. (2017). Treading on eggshells: ‘doing' feminism in educational research. Int. J. Res. Method Educ. 1, 46–58. 10.1080/1743727X.2017.1399354

[B48] Ruan N. (2020). Interviewing elite women professors: methodological reflections with feminist research ethics. Qual. Res. 1, 110–125. 10.1177/1468794120965368

[B49] Saraswati M. Beta A. R. (2021). Knowing responsibly: decolonizing knowledge production of Indonesian girlhood. Femin. Media Stud. 21, 758–774. 10.1080/14680777.2020.1763418

[B50] Taft J. K. (2010). Rebel Girls: Youth Activism and Social Change Across the Americas. New York City: NYU Press.

[B51] Taft J. K. (2014). The political lives of girls. Sociol. Compass 8, 259–267. 10.1111/soc4.12135

